# Supplementary material for: Complexome profiling of the Chlamydomonas psb28 mutant reveals TEF5 as an early PSII assembly factor
Source: Plant Cell. 2025 Mar 17;37(6):koaf055. doi: 10.1093/plcell/koaf055 (PMC12164590; doi:10.1093/plcell/koaf055)
Supplement: koaf055_Supplementary_Data [file koaf055_supplementary_data.zip › psb28_interactive.zip!/plots/intensity/qualityPlots/peptides/AOF1_Cre09.g389250.t1.2.html]

### Cre09.g389250.t1.2

Trivial name: AOF1  
SeqCoverage [median]: 12.40  
Mass: 48.82 kDa  
  
 

| Sequence | MissedCleavage | Mass [Da] | wtIntensity | muIntensity | Score |
| --- | --- | --- | --- | --- | --- |
| AALAAPVAGK | 0 | 867.52 | 774.59 | 0.00 | 55.68 |
| AATQITAVLSGGAAAAAPR | 0 | 1695.93 | 0.00 | 1267.51 | 43.95 |
| EKFPDVVVLEAQNR | 1 | 1642.87 | 0.00 | 1956.93 | 38.99 |
| GMIAFNPSLPAPK | 0 | 1341.71 | 525.28 | 0.00 | 62.82 |
|  |  |  | 1299.9 | 3224.4 |  |
